# Mesenchymal stem cells inhibit RANK-RANKL interactions between osteoclasts and Th17 cells *via* osteoprotegerin activity

**DOI:** 10.18632/oncotarget.21379

**Published:** 2017-09-28

**Authors:** Kyung-Ah Cho, Minhwa Park, Yu-Hee Kim, Kyung-Ha Ryu, So-Youn Woo

**Affiliations:** ^1^ Department of Microbiology, College of Medicine, Ewha Womans University, Seoul, Republic of Korea; ^2^ Department of Pediatrics, College of Medicine, Ewha Womans University, Seoul, Republic of Korea

**Keywords:** mesenchymal stem cells, osteoprotegerin, Th17 cells, osteoclasts, psoriasis, Immunology and Microbiology Section, Immune response, Immunity

## Abstract

Th17 cells play a critical role in several autoimmune diseases, including psoriasis and psoriatic arthritis (PsA). Psoriasis is a chronic inflammatory skin disease associated with systemic inflammation and comorbidities, such as PsA. PsA develops in nearly 70% of patients with psoriasis, and osteoclasts associated bone erosion is a hallmark of the disease. Thus far, the effect of Th17 cells on osteoclastogenesis *via* direct cell-to-cell interactions is less understood. In this study, we observed that Th17 cells directly promote osteoclast differentiation and maturation *via* expression of receptor activator of nuclear factor-κ β ligand (RANKL) *in vitro*. We investigated the impact of conditioned medium obtained from human palatine tonsil-derived mesenchymal stem cells (T-CM) on the interactions between osteoclasts and Th17 cells. T-CM effectively blunted the RANK-RANKL interaction between the osteoclast precursor cell line RAW 264.7 and Th17 cells *via* osteoprotegerin (OPG) activity. The frequency of tartrate-resistant acid phosphatase (TRAP)-positive cells in the bone marrow of an imiquimod (IMQ)-induced psoriasis mouse model was decreased following T-CM injection. Therefore, our data provide novel insight into the therapeutic potential of tonsil-derived mesenchymal stem cell-mediated therapy (*via* OPG production) for the treatment of pathophysiologic processes induced by osteoclasts under chronic inflammatory conditions such as psoriasis.

## INTRODUCTION

Inflammation is an immune defense against pathogens or danger signals. Persistent inflammatory responses result in chronic inflammatory diseases that are often associated with bone destruction, even when the inflammatory site is not the bone, such as in psoriasis [1–3]. Approximately a third of patients suffering from psoriasis develop psoriatic arthritis (PsA), a type of inflammatory arthritis marked by joint pain, swelling, and stiffness. In addition to the skin, PsA targets the spine, peripheral joints, and entheses, and can lead to destructive bone loss. Up to 70% of PsA patients exhibit signs of erosive bone disease [4].

Osteoclasts (OCL) are multinucleated cells from the monocyte/macrophage lineage that degrade bone matrix and dynamically remodel the skeleton. Bone remodeling is a highly regulated process involving complex interactions between bone-forming osteoblasts (OBL) and bone-resorbing OCLs [5]. These interactions require cell-to-cell contact, cytokine production, and coupling factor generation during bone resorption. OCL generation is supported by OBLs, which produce macrophage colony-stimulating factor (M-CSF) and receptor activator of nuclear factor- β ligand (RANKL), the two essential signals for OCL differentiation from myeloid cells such as inflammatory monocytes, macrophages, and dendritic cells [6, 7]. OBLs also negatively regulate OCL by producing osteoprotegerin (OPG), a protein that inhibits the development of OCL, acts as a decoy receptor by sequestering RANKL and inhibiting RANK signaling [8].

Systemic inflammation induces high levels of circulating inflammatory immune cells that can interact with bone cells through multiple pathways and that, in turn, synergize to cause OCL activation [9, 10]. Considering that Th17 cells are key players in the immunopathogenesis of psoriasis, we hypothesized that Th17 cells could promote osteoclastogenesis *via* direct stimulation of OCL precursors. Th17 cytokines, including IL-17 and TNF-α, directly or indirectly regulate osteoclastogenesis [11]. In particular, Th17 cells activates synovial fibroblasts to produce RANKL in rheumatoid arthritis (RA) [12]. However, the direct cell-to-cell effect of Th17 cells on OCLs is still poorly understood.

Mesenchymal stem cells (MSCs) are multi-potent, non-hematopoietic progenitor cells that were originally harvested from the bone marrow (BM). However, they can now be isolated from multiple organs [13]. The immunomodulatory and tissue regeneration properties of MSCs, together with their low immunogenic potential, make them promising therapeutics for severe refractory autoimmune diseases. We previously reported that human palatine tonsil-derived mesenchymal stem cells (T-MSCs) are attractive immune modulators because they act on various immune and non-immune cells [14, 15]. Further, T-MSCs migrate to damaged tissue sites and participate in tissue repair [16].

In the present study, we investigated whether Th17 cells directly promote osteoclastogenesis and whether the Th17-cell interactions with OCL precursor cells could be hindered by soluble mediators secreted by T-MSCs. Thus, we assessed the therapeutic effects of conditioned medium from T-MSCs (T-CM) on the interaction of Th17 cells and OCL precursor cells *in vitro* and *in vivo*. OPG was determined to be a crucial mediator by which T-CM modulates Th17 cell-induced osteoclastogenesis.

## RESULTS

### Th17 cells express RANKL and promote osteoclast maturation

**Figure 1:**
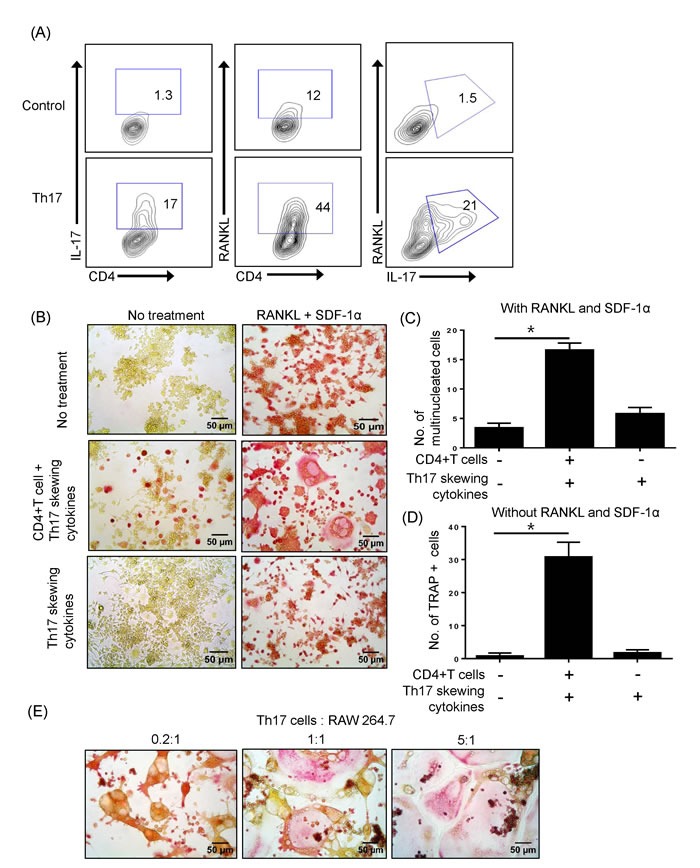
Th17 cells expressing RANKL promote osteoclast maturation **A.** Naïve CD4+ T cells were differentiated into Th17 cells for 5 days and the surface expression of RANKL and CD4 and the intracellular expression of IL-17 were detected by flow cytometry. **B.** The osteoclast precursor cell line RAW 264.7 was cultured with or without RANKL (50 ng/ml) and SDF-1a (20ng/ml) for 3 days. Naïve CD4+ T cells were added to the cultures with or without Th17-skewing cytokines (IL-6, IL-23, and TGF-β). After 3 days, RAW 264.7 cells were fixed and stained for TRAP activity. Total TRAP-positive cells in the absence of RANKL and SDF-1α **C**. TRAP-positive multinucleated cells in the presence of RANKL and SDF-1α were counted. **D**. Data are presented as means ± SEM (*P < 0.05). **E.** Th17 cells induced giant osteoclasts in a dose-dependent manner.

We investigated whether CD4^+^ T cells express RANKL upon differentiation into Th17 cells. As shown in Figure 1A, surface expression of RANKL was dramatically increased in IL-17+ Th17 cells. This prompted us to test whether Th17 cells directly influence osteoclast precursors. The osteoclast precursor cell line RAW 264.7 was cultured with or without CD4+ T cells in the presence of Th17-skewing cytokines (IL-6, IL-23, and TGF-β). On day 3, RAW 264.7 cells were stimulated with RANKL and SDF-1 and were TRAP-positive (Figure 1B, upper panel). Notably, RAW 264.7 cells co-cultured with T cells that underwent differentiation into Th17 cells formed multinucleated cells after only 3 days (Figure 1B, middle panel). To exclude the direct effect of Th17-skewing cytokines, RAW 264.7 cells were treated with those cytokines in the absence of T cells. In this scenario, IL-6, IL-23, and TGF-β did not increase TRAP staining or the number of multinucleated cells (Figure 1B, lower panel). Surprisingly, Th17 cells, even in the absence of RANKL and SDF-1α, induced TRAP staining in RAW 264.7 cells, indicating that Th17 cells alone are potentially sufficient to promote osteoclastogenesis (Figure 1B, middle panel). Those TRAP+ cells or TRAP+ multinucleated cells were counted in the 200 X field using a phase-contrast microscope and showed significant increase (Figure 1C-1D). Th17 cells augmented the formation of giant osteoclasts in a dose-dependent manner in cocultures with various Th17 cell to RAW 264.7 cell ratios (Figure 1E).

### T-MSCs constitutively produce osteoprotegerin (OPG)

**Figure 2:**
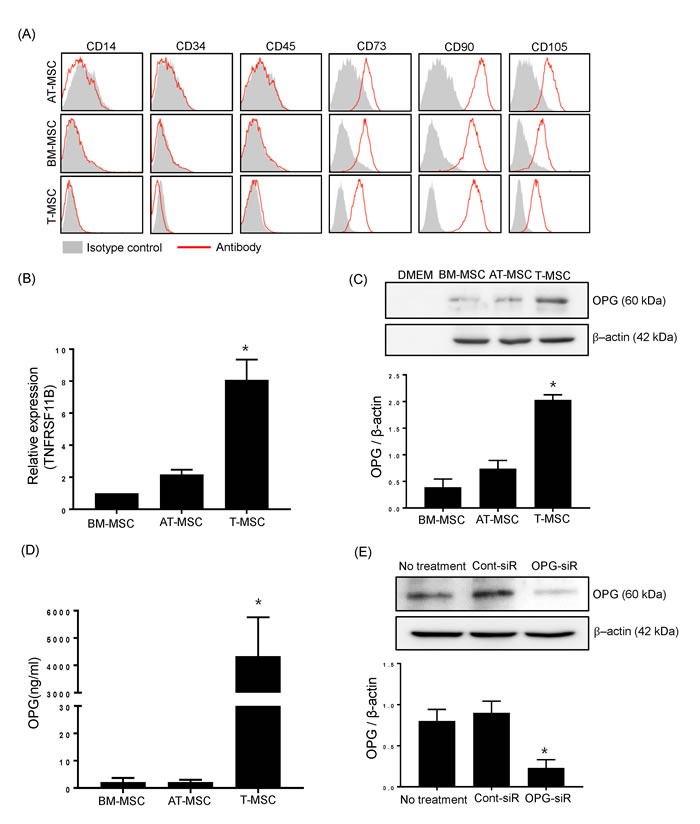
T-MSCs constitutively produce OPG **A.** The expression of surface antigens on BM-MSCs, AT-MSCs, and T-MSCs were detected by flow cytometry. Cells were negative for hematopoietic cell markers (CD14, CD34, CD45) and positive for CD73, CD90 and CD105. The data show a representative histogram from three experiments. **B.** BM-MSCs, AT-MSCs, and T-MSCs were harvested and the mRNA expression of TNFRSF11B (OPG encoding gene) was analyzed by real time-quantitative PCR. Data are presented as means ± SEM (*P < 0.05). **C.** Cell culture supernatants were collected and subjected to western blotting to detect secreted OPG from BM-MSCs, AT-MSCs, and T-MSCs. The pixel densities of the OPG bands were divided by those of the corresponding β-actin bands for normalization. Data are presented as means ± SEM (*P < 0.05). **D.** The OPG levels in cell supernatants from BM-MSCs, AT-MSCs, and T-MSCs were measured by ELISA. Data are presented as means ± SEM (*P < 0.05). **E.** *Opg*-specific siRNA effectively knocked down endogenous OPG expression in T-MSCs. The pixel densities of the OPG bands were divided by those of the corresponding β-actin bands for normalization. Data are presented as means ± SEM (*P < 0.05).

We investigated candidate soluble factors produced by T-MSCs that could modulate Th17 cell-induced osteoclastogenesis. OPG, a protein that inhibits the development of osteoclasts, acts as a decoy receptor by sequestering RANKL and inhibiting RANK signaling [8]. OPG is located on the bone surface under osteoclasts to prevent excessive resorption. However, OPG is expressed not just in bone, but in many cell systems and tissues. Thus, we further investigated whether T-MSCs constitutively express OPG. Besides T-MSCs, BM-MSCs and AT-MSCs which determined their specific surface antigen are also tested for detection of OPG (Figure 2A). Quantitative RT-PCR analysis indicated that T-MSCs expressed significantly higher levels of OPG compared to BM-MSCs and AT-MSCs (Figure 2B). T-MSCs likely constitutively produced OPG because the T-MSC-cell culture supernatant contained significantly higher levels of OPG compared to supernatants from cultures of the other types of MSCs (BM-MSCs and AT-MSCs) (Figure 2C). For further quantitative measurement of OPG secretion, we performed ELISA analysis to detect human OPG in cell supernatant from BM-MSCs, AT-MSCs, and T-MSCs. T-MSCs shown to produce abundant OPG in comparison with BM-MSCs AT-MSCs (Figure 2D). To confirm the effects of OPG on Th17 cell-mediated osteogenesis, we generated OPG-knockdown T-CM using OPG-specific siRNA (Figure 2E). The OPG levels in OPG-knockdown T-CM were reduced up to 80% following siRNA transfection. These OPG-knockdown T-CM were used parallel with normal control T-CM in treating Th17 cells - RAW 264.7 cells coculture.

### T-MSCs inhibit interactions between Th17 cells and osteoclasts in an OPG-dependent manner

**Figure 3:**
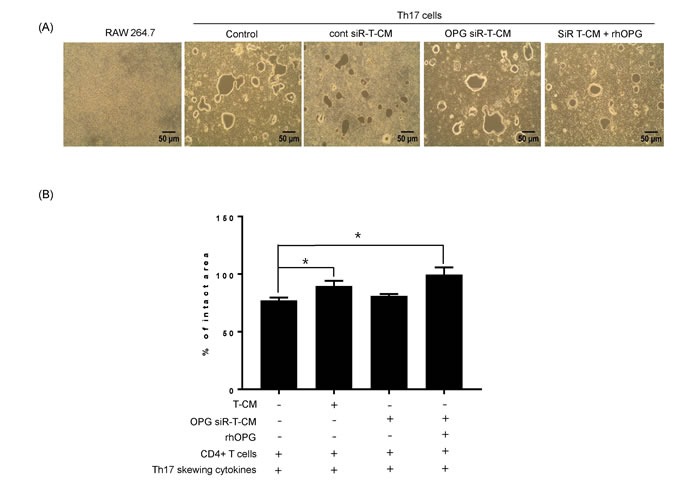
Th17 cells induce osteoclastogenesis in the absence of RANKL stimulation **A.** RAW 264.7 cells were cultured on calcium phosphate-coated plates. Naïve CD4+ T cells were added to the RAW 264.7 cell cultures with anti-CD3 Ab, anti-CD28 Ab, IL-6, IL-23, and TGF-β. To test the effect of T-MSC-derived OPG, RAW 264.7 cell cultures were treated with naïve CD4+ T cells and T-CM, OPG-knockdown T-CM, control siRNA knockdown T-CM, or OPG-knockdown T-CM supplemented with rhOPG. After 6 days, resorption activity assays were performed. **B.** Intact pit areas were assessed using ImageJ software. Data are presented as means ± SEM (*P < 0.05).

Based on our findings for the role of Th17 cells in the early stage of osteoclastogenesis, we hypothesized that Th17 cells would also augment the late stage of RANKL-mediated osteoclast differentiation. Thus, we assessed the capacity of osteoclasts to resorb a mineralized matrix under various experimental conditions and whether Th17 cells support osteoclast maturation. RAW 264.7 cells supplemented with Th17 cells without RANKL and SDF-1α stimulation formed bone resorption pits on the bone matrix plate (Figure 3A). We determined the functional implications of T-MSC inhibition on the osteoclastogenesis augmented by Th17 cells. T-CM suppressed osteoclast activity in the presence of Th17 cells, and exogenous rhOPG treatment rescued the suppressed activity in OPG-knockdown T-CM. When the percentage of intact pit area on RAW 264.7 cell cultured group determined as 100%, intact pit area in other groups showed significant difference as shown in the Figure 3B.

**Figure 4:**
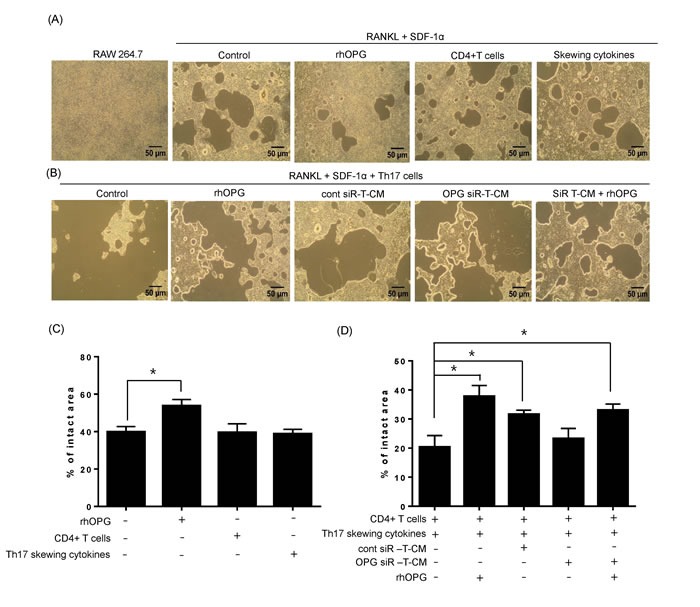
Th17 cells enhance osteoclast activity in the presence of RANKL stimulation **A.** RAW 264.7 cells were cultured on calcium phosphate-coated plates and stimulated with RANKL (50 ng/ml) and SDF-1 (20 ng/ml). Recombinant rhOPG (100 ng/ml), naïve CD4+ T cells (with anti-CD3 and anti-CD28 Abs), or Th17-skewing cytokines alone (IL-6, IL-23, and TGF-β) were added. **B.** RAW 264.7 cells were cultured with Th17 cells in the presence of RANKL (50 ng/ml) and SDF-1 (20 ng/ml). OPG-knockdown T-CM or control siRNA-treated T-CM were added to RAW 264.7 cell cultures to observe the effect of T-MSC-derived OPG. Exogenous rhOPG was used as a positive control and was also added to OPG-knockdown T-CM. **C.**-**D.** After 6 days, resorption activity assays were performed and the intact pit areas were assessed using ImageJ software. Data are presented as means ± SEM (*P < 0.05).

Next, we examined osteoclast activity under normal differentiation conditions that included RANKL. As shown in Figure 4A, numerous large bone resorption pits were formed on the bone matrix plate in the presence of RANKL and SDF-1. CD4+ T cells or Th17-skewing cytokines did not affect the formation of bone resorption pits, whereas, rhOPG treatment strongly diminished bone resorption pits resulting from RANKL and SDF-1-induced osteoclast maturation. When osteoclasts encountered differentiated Th17 cells, the density of bone resorption pits was dramatically increased. T-CM significantly reduced the size of pit areas, even in the presence of Th17 cells, and was as effective as the rhOPG treatment. The T-CM from OPG-knockdown T-MSCs had decreased effects on the regulation of osteoclast activity, but supplementation with rhOPG rescued the activity, suggesting a direct role for OPG (Figure 4B). Those bone resorptive activities were statistically compared by assessing intact pit area of bone matrix plate in each experimental group (Figure 4C-4D).

### T-MSCs decreased skin inflammation with TRAP positive cells in a mouse psoriasis model

To further test the suppression of osteoclast activation that occurs in systemic inflammatory condition such as psoriasis, we injected T-CM into mice with IMQ-induced psoriasis-like dermatitis. T-CM was administered on the first and third days of the 6 days of IMQ application.

Considering that the result of quantification of OPG in ELISA analysis, we assumed that the approximate amount of OPG in the given volume (200 ul/mouse) is 900 ng.

**Figure 5:**
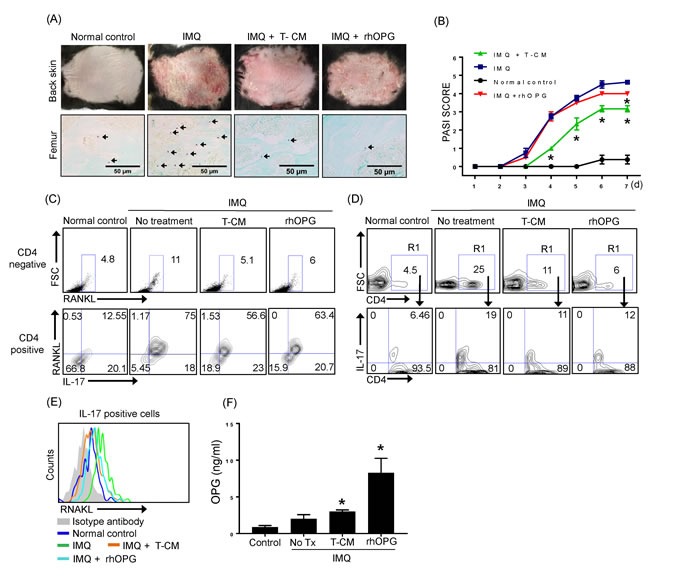
IMQ-induced skin inflammation and the frequency of TRAP+ cells in bone marrow were reduced following T-CM injection in a psoriasis mouse model **A.** Back skin of normal control mice, IMQ-treated mice, IMQ-treated mice injected with T-CM, and rhOPG-injected mice displayed different grades of erythema, scaling, and thickening (upper panel). Photographs were taken on the seventh day after application of IMQ for 6 consecutive days. TRAP+ cells in femurs from normal control mice, IMQ-treated mice, IMQ-treated mice injected with T-CM, and rhOPG-injected IMQ mice were detected by TRAP staining (lower panel). **B.** Erythema, scaling, and thickness of back skin was scored daily on a scale from 0 to 4. Cumulative scores are depicted. Data are presented as means ± SEM (*P < 0.05). **C.** RANKL expressing cells in bone marrow from normal control mice, IMQ-treated mice, IMQ-treated mice injected with T-CM, and rhOPG-injected mice were detected by flow cytometry. Plots from flow cytometric analyses show the percentage of CD4^-^ RNAKL^+^ cells in each experimental group (upper panel) and CD4^+^IL-17^+^RANKL^+^ cells (lower panel). **D.** CD4^+^ IL-17^+^ Th17 cells in peripheral blood were detected by flow cytometry. The results shown are representative of three independent experiments. **E.** RANKL expression on IL-17 gated cells in each experimental group are shown as representative histogram by flow cytometric analysis. **F.** The levels of OPG in bone marrow fluid in normal control mice, IMQ-treated mice, IMQ mice injected with T-CM, and IMQ mice injected with rhOPG were quantified by ELISA. Data are presented as means ± SEM (*P < 0.05).

After 6 days of IMQ application, the back skin of normal control mice, IMQ mice, T-CM-injected IMQ mice, and IMQ mice injected with rhOPG displayed significantly different grades of erythema, scaling, and thickness as determined by PASI scoring (Figure 5A-5B). Control mice that were shaved and treated daily with control cream did not show any signs of inflammation. The severity of the skin lesions (erythema, scaling, and thickness) was relieved in IMQ mice injected with T-CM (Figure 5A, upper panel). Additionally, the frequency of TRAP-positive cells in femur BM was increased in the IMQ-treated mice, indicating that such skin inflammation may be linked to osteoclast activation in the bone. Mice treated with rhOPG did not show significant reductions in their skin psoriatic symptoms; however, rhOPG treatment reduced the number of TRAP-positive cells in the femurs of psoriatic mice. The frequency of TRAP-positive cells was decreased in T-CM-injected IMQ mice in comparison with the mice treated with IMQ alone (Figure 5A, lower panel). As expected, the frequency of RANKL expressing cells in BM was increased in the IMQ mice, which may be a cause of the increase in TRAP-positive cells. When we divide the percentage of RANKL expressing cells based on the expression of CD4 in BM, we observed increase of RANKL expressing cells on CD4 negative fractions as well as positive fraction. In particular, IL-17^+^RANKL^+^ cells on CD4 positive gating dramatically increased (Figure 5C). The Th17 cells, the major cell source for RANKL in our scenario are shown to extended into circulation as revealed by the increase in peripheral blood in IMQ mice. However, T-CM or rhOPG effectively downregulate the peripheral increase of Th17 cells (Figure 5D). The expression of RANKL on IL-17 positive cells in peripheral blood was also increased under IMQ-induced inflammatory status, but reduced by injection of T-CM or rhOPG (Figure 5E). We previously demonstrated that T-MSCs effectively inhibit Th17 differentiation [17]. The present result coincides with those earlier in the study because T-CM injection reduced RANKL^+^IL-17^+^CD4^+^ cells in both BM and peripheral blood.

Lastly, we examined the levels of OPG in BM fluid from each experimental mice. Although baseline level of OPG is shown to be elevated in BM under inflammatory condition in IMQ mice, OPG supplementation seems to be critical to reduce OCL activation as revealed by significant increase of OPG in T-CM injected IMQ mice (Figure 5F).

## DISCUSSION

In this study, we demonstrated that conditioned medium from T-MSCs (T-CM) can regulate Th17 cell-induced osteoclastogenesis *via* OPG, a soluble protein that inhibits production of OCLs. Th17 cells alone were sufficient to enhance osteoclast differentiation and activation by expressing RANKL in coculture with osteoclast precursor cells, whereas T-CM efficiently inhibited osteoclastogenesis by reducing the interaction between Th17 cells and osteoclast precursor cells. Furthermore, the frequency of TRAP+ cells in BM was decreased following T-CM injection in a psoriatic dermatitis mouse model.

Dysregulated osteoclastogenesis and production of tissue-degrading molecules in response to inflammatory stimuli result in the destruction of cartilage and bones. The chronic inflammatory skin disease psoriasis carries an increased risk of comorbidities that include PsA. RANK-mediated osteoclastogenesis has been implicated in the pathogenesis of bone resorption in PsA. In patients with PsA, osteoclasts are present at sites of bone erosion, and osteoclasts cultured *in vitro* from peripheral blood precursors exhibit increased resorptive activity compared with those from healthy controls [18]. Intense RANKL expression has been demonstrated within the lining layer in PsA joints, with more restricted OPG expression in the sublining layer, which implicates an imbalance in the RANKL/OPG axis that, in turn, may promote osteoclastogenesis [18]. Chronic inflammatory milieu in psoriasis appears to be high risk factor to disturb RANKL/OPG axis that ultimately lead to osteoclasts activation.

Thus far, studies of CD4+ helper T cells involved in osteoclastogenesis have predominantly focused on the role of IL-17+ Th17 cells [12]. IL-17 is associated with increased osteoclastogenesis in chronic inflammation and promotes cartilage destruction and bone erosion by inducing RANKL expression on OBLs [19, 20]. Although it is recognized that osteoclastogenesis is triggered by Th17 T cells, those findings were restricted to an investigation of the indirect role mediated by OBLs. Here, we identified a potential link between Th17 cells and osteoclasts through direct cell-to-cell interactions.

MSCs contribute to tissue regeneration by modulating inflammation during stem cell treatment of inflammatory diseases [21, 22]. A chronic inflammatory status can eventually cause the destruction of target organ tissue in accordance with the specific disease. Therefore, stem cell therapy using MSCs or its conditioned medium could be an effective therapeutic strategy for the treatment of many chronic inflammatory diseases that are accompanied by tissue injury. Because psoriasis is a chronic, relapsing, autoimmune disease that could affect the spine, peripheral joints, entheses, and skin tissue, MSC-based cytotherapy may be a promising therapeutic option.

Tonsil-derived mesenchymal stem cells (T-MSCs) inhibit differentiation and proliferation of dendritic cells (DCs), B cells, and T cells *in vitro* [15, 23]. In particular, the T-MSC-derived immunomodulatory protein PD-L1 efficiently inhibits Th17 differentiation from CD4+ naïve T cells [17]. In the context of the pathologic roles of Th17 cells in the psoriatic condition, we hypothesized that T-MSCs modulate the interactions of Th17 cells with other cells, including osteoclasts. Here, Th17 cells alone were capable of enhancing differentiation and maturation of osteoclasts by expressing RANKL. The cytokines that induce Th17 differentiation (IL-6, IL-23, and TGF-β) were not sufficient to induce osteoclastogenesis without RANKL. Furthermore, those was no additive effect of Th17 skewing cytokines, even in the presence of RANKL. These results indicate that osteoclast activation is primarily dependent on RANKL signalling rather than an inflammatory milieu. Thus, our findings provide new insight as to how Th17 cells may be a potent cell source for promoting comorbidity such as PsA *via* direct interactions with osteoclasts.

OPG is mainly produced by osteoblasts and generates a negative feedback loop by inhibiting RANKL-RANK ligation [8, 24]. Our results suggest that T-MSCs constitutively produce significantly higher levels of OPG compared to other sources, such as BM- and AT-MSCs. Palatine tonsils are a secondary lymphoid tissue that continuously encounter antigens and subsequently drive efficient immune responses [25, 26]. Thus, those tissues may specifically render intrinsic priority to T-MSCs in terms of immune regulatory properties. We demonstrated that T-CM suppressed the pathophysiologic interactions of Th17 cells and osteoclasts *via* OPG activity. Likewise, TRAP+ cells in BM were present in a psoriatic skin dermatitis mouse model and their frequency was reduced following T-CM injection. In general, OPG is produced by osteoblasts to maintain bone homeostasis. However, pathophysiologic circumstances may overwhelm the usual negative feedback loop causing a slant toward osteoclastogenesis. Thus, OPG supplementation may be a critical key for resolving the bone erosion that occurs in the inflammatory bone microenvironment. Because T-MSCs stably express OPG, T-MSCs and T-CM are good sources of OPG for use in the treatment of inflammatory diseases that involve OCL activation such as PsA.

In conclusion, the present study suggests a novel role for Th17 cells in OCL activation and suggests therapeutic options through the following insights. First, Th17 cells are professional osteoclastogenic cells due to their expression of RANKL. Second, T-MSCs constitutively produce high quantities of OPG that effectively blunt the activity of RANKL. Therefore, T-MSCs or T-CM may be a promising therapeutic agent for targeting the inflammation associated with OCL.

## MATERIALS AND METHODS

### Ethics statement and human samples

Human tonsils were obtained from tonsillectomies performed in the Department of Otorhinolaryngology, Head and Neck Surgery at Ewha Womans University Mok-Dong Hospital. The protocol was approved by the research ethics committees of Ewha Womans University Mok-Dong Hospital (IRB #ECT11-53-02). Written informed consent was obtained from all patients. Female C57BL/6 mice at 8 weeks of age were purchased from OrientBio (Emsung, Seoul, Korea). All animals were maintained under pathogen-free conditions on a 12-hr light/dark cycle with free access to food and water. All procedures were approved by the Animal Care and Use Committee of the Ewha Womans University School of Medicine (ESM 15-0309) and all experiments were performed in accordance with the approved guidelines and regulations.

### Th17 cell differentiation

Mouse naïve CD4^+^ T cells are isolated by depletion of memory CD4^+^ T cells and non-CD4^+^ T cells from single-cell suspensions of C57BL/6 female mouse spleen and draining lymph nodes *via* magnetic isolation using naive CD4^+^ T cell isolation kit (Miltenyi Biotec, GmbH, Gladbach, Germany). Single-cell suspensions of CD4^+^ naïve cells were seeded at a density of 2×10^5^ cells per well in 96-well plates with RPMI medium containing 10% fetal bovine serum (FBS), 1% penicillin/streptomycin solution, 2 mM L-glutamine, and 50 μM 2-mercaptoethanol. To induce differentiation into Th17 cells, cells were stimulated with anti-CD3 antibody (2.5 g/ml, Biolegend, San Diego, CA, USA), anti-CD28 antibody (5 μg/ml, Biolegend), IL-6 (50 ng/ml, Biolegend), IL-23 (20 ng/ml, Biolegend), TGF-β (5 ng/ml, Biolegend), and anti-IFN-γ blocking antibody (100 ng/ml, Biolegend) for 6 days.

### Flow cytometry

To characterize immunotypes on BM-MSCs, AT-MSCs, and T-MSCs, cells were stained with the following antibodies: PE-conjugated anti-human CD14 (M5E2, Mouse IgG_2a_; BD Biosciences, Franklin Lakes, NJ, USA), PE-conjugated anti-human CD34 (2D1, Mouse IgG_1_; BD Biosciences), PerCP-conjugated anti-human CD45 (581, Mouse IgG_1_; BD Biosciences), PE-conjugated anti-human CD73 (AD2, Mouse IgG_1_; BD Biosciences), PE-conjugated anti-human CD90 (5E10, Mouse IgG_1_; BD biosciences), FITC-conjugated anti-human CD105 (5N6, Mouse IgG_1_; SEROTEC, Kidlington, UK). Nonspecific level was determined using PE-conjugated anti-mouse IgG_2a_ (X39, BD Biosciences), PerCP-conjugated anti-mouse IgG_1_ (X40, BD Biosciences), PE-conjugated anti-mouse IgG_1_ (X40, BD Biosciences), FITC-conjugated anti-mouse IgG_1_ (RMG1-1, Biolegend) antibodies.

The surface expression of RANKL and CD4 on Th17 cells was detected by labeling differentiated murine Th17 cells with PE-conjugated anti-mouse RANKL (IK22/5, Biolegend) and PerCP-conjugated anti-mouse CD4 (GK1.5, Biolegend) antibodies. Intracellular levels of IL-17 in Th17 cells were detected by staining with Alexa Fluor 488-conjugated anti-mouse IL-17 (TC11-18H10.1, Biolegend). Each sample was acquired on a FACSCalibur system (BD Biosciences) and the data were analyzed using FlowJo software (Tree Star, Ashland, OR, USA). For the detection of CD4^+^IL-17^+^RANKL^+^ cells in BM from experimental mice, cells were similarly stained using same antibodies. In other hands, CD4 + IL-17+ cells in peripheral blood were labeled with PE- conjugated anti-mouse CD4 (GK 1.5, Biolegend) and PerCP-conjugated anti-mouse IL-17 (TC11-18H10.1, Biolegend) antibodies. Additionally, RANKL expressing cells on IL-17^+^ cells in peripheral blood were detected by staining with PE-conjugated anti-mouse RANKL (IK22/5, Biolegend) antibody.

### Osteoclastogenesis

RAW 264.7 cells were cultured at a density of 2×10^5^ cells/well in 6-well plates in DMEM containing 10% FBS and antibiotics (10,000 U/ml penicillin, 10 mg/ml streptomycin, and 25 μg/ml amphotericin B in 0.85% NaCl solution) in the presence of 50 ng/ml recombinant mouse RANKL (R&D Systems, Minneapolis, MN, USA) and 20 ng/ml recombinant mouse SDF-1α (R&D Systems). SDF-1 α was replenished daily on the first 2 days during the initial stage of differentiation, while RANKL was replenished once every two days for 6 days. Medium was changed every 2 days. Cultures were maintained at 37°C in a humidified 5% CO_2_ atmosphere.

### TRAP staining of cultured adherent cells

RAW 264.7 cells were fixed with 10% glutaraldehyde for 15 min at 37°C. After washing twice with phosphate-buffered saline (PBS) pre-warmed to 37°C, cells were treated with TRAP staining solution (0.2 M sodium acetate, 0.2 M acetic acid, 0.3 M sodium tartrate, 10 mg/ml phosphate disodium salt, 0.1% Triton X-100, and 0.3 mg/ml Fast Red Violet LB (F-3381, Sigma Aldrich, St Louis, MO, USA) in distilled water) for 10 min at 37°C. TRAP-positive cells appeared dark red. TRAP-positive multinucleated cells containing three or more nuclei were counted as OCLs.

### TRAP staining of mouse bone samples

Paraffin-embedded mouse femur sections were deparaffinized and rehydrated through graded ethanol to distilled water. Slides containing the deparaffinized and rehydrated sections were placed in pre-warmed TRAP staining solution and incubated at 37°C for 30 min. After rinsing with distilled water, slides were counterstained with 0.02% Fast Green (Sigma Aldrich) for 30 sec and rinsed with distilled water. Slides were rapidly dehydrated through graded ethanol at 5-sec increments.

### Preparation of conditioned medium

T-MSCs were obtained and maintained as previously reported [27]. T-MSCs used in this study were from tonsillectomy performed on male at age 10. AT-MSCs were generously provided by RNL Bio (Seoul, Korea) and BM-MSCs were purchased from the Severance Hospital Cell Therapy Center (Seoul, Korea). To generate MSC-conditioned medium (MSC-CM), BM-derived MSCs (BM-MSCs), adipose tissue-derived MSCs (AT-MSCs), and T-MSCs (at passages 7-8) were grown to 80-90% confluency in 100-mm tissue culture plates. Cells were washed twice with PBS and the medium was replaced with serum-free DMEM. Medium was collected after 48 h of culture, centrifuged at 1300 rpm for 5 min, and passed through 0.2-μm filters to generate CM. CM was concentrated 20-fold by centrifugal filtration (3K cut-off; Amicon Ultra-15, Millipore, Billerica, MA, USA). Concentrated CM was frozen and stored at -80°C for future use. Serum-free culture medium was processed in the same manner to serve as a negative control.

### Quantitative reverse transcription PCR (qRT-PCR)

To analyze the expression of TNFRSF11B, the OPG encoding gene, BM-MSCs, AT-MSCs, and T-MSCs were harvested and homogenized in TRIzol^®^ (Invitrogen, Carlsbad, CA, USA). Complementary DNA was synthesized using First-Strand cDNA Synthesis Kits (Toyobo, Osaka, Japan) according to the manufacturer's instructions. Real time PCR analysis was performed on a StepOnePlus instrument (Applied Biosystems) using SYBR green (TOYOBO). TNFRSF11B (OPG, 226 bp) was amplified using the primers: 5’-GCGCTCGTGTTTCTGGACA-3’ (forward) and 5’- AGTATAGACACTCGTCACTGGTG -3’ (reverse). The internal control gene GAPDH (192 bp) was amplified using the primers: 5’-GGT AAA GTG GAT ATT GTT GCC ATC AAT G-3’ (forward) and 5’-GGA GGG ATC TCG CTC CTG GAA GAT GGT G-3’ (reverse). The expression of TNFRSF11B was normalized to the expression of GAPDH and Relative fold expression and changes were calculated using the 2^-ΔΔCt^ method.

### Immunoblot

Equal amounts (20 μl per lane) of CM from the different types of MSCs (BM-MSCs, AT-MSCs, and T-MSCs) were subjected to western blotting to compare the extent of OPG released into the cell culture supernatant. OPG expression was detected using anti-OPG Ab (H-249, Santa Cruz Biotechnology, Santa Cruz, CA, USA). As an internal control, β-actin expression in the different types of MSCs was also determined by western blot using anti-β-actin (C4, Santa Cruz Biotechnology). The band pixel densities of OPG were divided by the pixel densities of the corresponding β-actin bands for quantitation using UN-SCAN-IT-gel 6.1 software. For the detection and normalization of intracellular OPG after transfection, expression of the OPG and housekeeping gene β-actin were detected using same antibodies used above.

### ELISA

To quantify OPG secretion from BM-MSCs, AT-MSCs, and T-MSCs, conditioned medium was collected and the levels of secreted OPG were determined using human OPG ELISA kit in accordance with the manufacturer's recommended protocols (Cat.No. E-EL-H1341, Elabscience Biotechnology Co., Wuhan, Hubei, China). In other hands, the levels of OPG in BM fluid from experimental mice (normal control mice, IMQ mice, T-CM injected IMQ mice, and OPG injected IMQ mice) were measured using mouse OPG ELISA kit in accordance with the manufacturer's instuctions (Cat.No. SEA108Mu, Cloud-Clone Corp., Houston, TX, USA).

### Transfection

To reduce endogenous OPG expression, T-MSCs were transfected with OPG-specific siRNA oligonucleotides (Santa Cruz Biotechnology) using Lipofectamine® 2000 reagent (Thermo Fisher Scientific, Waltham, MA, USA) in accordance with the manufacturer's instructions. Non-targeted siRNA oligonucleotides (Santa Cruz Biotechnology) were used as negative controls. At 48-h post-transfection, cells were harvested for protein extraction and 10 μg of protein was used to confirm OPG knockdown by western blotting.

### Bone resorption activity assay

To assess resorption activity of OCLs, RAW 264.7 cells (5×10^4^ cells/well) were plated in calcium phosphate-coated 24-well plates [Osteoclast Activity Assay Substrate (OAAS); Cosmo Bio, Tokyo, Japan] in the presence of RANKL (50 ng/ml) and SDF-1α (20 ng / ml). To test the effect of Th17 cells on OCL activity, Th17 cells (10^4^ cells/well) were added with or without recombinant human OPG (rhOPG, 100 ng/ml, R&D Systems). To test the effect of T-CM on the Th17 cell-OCL interaction, T-CM (20 μl) or OPG knocked-down T-CM was added to OCL cultures. Exogenous rhOPG supplementation (100 ng/ml) in the OPG knocked-down T-CM treatment group was also performed. Non-differentiated CD4+ T cells or a Th17-skewing cytokine cocktail (IL-6, IL-23, and TGF-β) was added to confirm their potential effect on OCL activity. After 6 days of culture, medium was removed, plates were washed with PBS, and cells were incubated with 5% sodium hypochlorite solution for 10 min to detach cells. After washing with PBS three times, plates were dried completely and resorption areas were observed by contrast microscopy. Pit areas were measured using ImageJ software (https://imagej.nih.gov/ij/).

### Mice and treatments

To induce skin inflammation in C57BL/6 mice using the inflammation response modifier drug imiquimod (IMQ), female C57BL/6 mice at 8 weeks of age received a daily topical dose of 83 mg of commercially available IMQ cream (5%, Aldara; Dong-Ah Pharmaceutical, Seoul, Korea) for 6 consecutive days. Normal control mice were treated similarly with a control vehicle cream (Vaseline Lanette cream, Fargon, The Netherlands). The effect of T-CM was tested by administration of 200 μl T-CM containing approximately 900 ng of OPG *via* the mouse tail vein on days 1 and 3 of the IMQ application period. Control mice received an intravenous injection of an equal volume of PBS *via* tail vein at the same time points. Additionally, rhOPG (1 μg/mouse) was given *via* tail vein to select treatment groups on days 1 and 3 of the IMQ application period.

### Scoring the severity of skin inflammation

An objective scoring system based on the clinical Psoriasis Area and Severity Index (PASI) was applied to score the severity of inflammation of the back skin. Thus, erythema, scaling, and thickening were scored independently on a scale from 0 to 4 as follows: 0, none; 1, slight; 2, moderate; 3, marked; 4, very marked. The cumulative score served as a measure of the severity of inflammation (scale 0-12).

### BM cells and fluid preparation

BM was flushed from the medullary cavities of murine femurs from normal control mice, IMQ-treated mice, IMQ mice injected with T-CM, and IMQ mice injected with rhOPG with 2.5 ml of serum-free RPMI-1640 (Gibco BRL, Carlsbad, CA, USA) medium using a 25-G needle, filtered, and centrifuged at 330 × g for 5 min. The supernatant was collected for ELISA analysis. Erythrocytes were removed by incubation with ammonium-chloride-potassium (ACK) lysis buffer. Cells were washed twice in PBS containing 0.5% FBS and 0.1% sodium azide and used for flow cytometry.

### Peripheral blood cell isolation

For peripheral blood collection, mice were anesthetized by injection with a mixture of Zoletil (30 mg/kg, Virbac Laboratories, Carros, France) and Rompun (10 mg/kg, Bayer, Leverkusen, Germany). Blood was collected through cardiac puncture and erythrocytes were removed by incubation with ACK lysis buffer. Cells were washed twice in FACS buffer (10% FBS, 10 mM EDTA, 20 mM HEPES, 1mM sodium pyruvate, 10 U/ml penicillin, 10 ug/ml streptomycin) and used for flow cytometry.

### Statistical analysis

Data are presented as means ± standard error of the mean (SEM). Statistical significance was determined by One way ANOVA or Student's *t*-test using the GraphPad Prism 7 software (GraphPad Software Inc.). For all analyses, *P* < 0.05 was considered statistically significant.

## References

[1] D’Epiro S, Marocco C, Salvi M, Mattozzi C, Luci C, Macaluso L, Giancristoforo S, Campoli M, Scarno M, Migliaccio S, Calvieri S, Richetta A (2014). Psoriasis and bone mineral density: implications for long-term patients. J Dermatol.

[2] Uluckan O, Wagner EF (2016). Role of IL-17A signalling in psoriasis and associated bone loss. Clin Exp Rheumatol.

[3] Uluckan O, Jimenez M, Karbach S, Jeschke A, Grana O, Keller J, Busse B, Croxford AL, Finzel S, Koenders M, van den Berg W, Schinke T, Amling M (2016). Chronic skin inflammation leads to bone loss by IL-17-mediated inhibition of Wnt signaling in osteoblasts. Sci Transl Med.

[4] Sakkas LI, Bogdanos DP (2016). Are psoriasis and psoriatic arthritis the same disease? The IL-23/IL-17 axis data. Autoimmun Rev.

[5] Boyce BF, Rosenberg E, de Papp AE, Duong LT (2012). The osteoclast, bone remodelling and treatment of metabolic bone disease. Eur J Clin Invest.

[6] Roodman GD (2006). Regulation of osteoclast differentiation. Ann N Y Acad Sci.

[7] Wakkach A, Mansour A, Dacquin R, Coste E, Jurdic P, Carle GF, Blin-Wakkach C (2008). Bone marrow microenvironment controls the in vivo differentiation of murine dendritic cells into osteoclasts. Blood.

[8] Perez-Sayans M, Somoza-Martin JM, Barros-Angueira F, Rey JM, Garcia-Garcia A (2010). RANK/RANKL/OPG role in distraction osteogenesis. Oral Surg Oral Med Oral Pathol Oral Radiol Endod.

[9] Matt P, Lindqvist U, Kleinau S (2015). Up-regulation of CD64-expressing monocytes with impaired FcgammaR function reflects disease activity in polyarticular psoriatic arthritis. Scand J Rheumatol.

[10] Mulligan JK, Bleier BS, O’Connell B, Mulligan RM, Wagner C, Schlosser RJ (2011). Vitamin D3 correlates inversely with systemic dendritic cell numbers and bone erosion in chronic rhinosinusitis with nasal polyps and allergic fungal rhinosinusitis. Clin Exp Immunol.

[11] Ciucci T, Ibanez L, Boucoiran A, Birgy-Barelli E, Pene J, Abou-Ezzi G, Arab N, Rouleau M, Hebuterne X, Yssel H, Blin-Wakkach C, Wakkach A (2015). Bone marrow Th17 TNFalpha cells induce osteoclast differentiation, and link bone destruction to IBD. Gut.

[12] Kim KW, Kim HR, Kim BM, Cho ML, Lee SH (2015). Th17 cytokines regulate osteoclastogenesis in rheumatoid arthritis. Am J Pathol.

[13] Liu S, Zhou J, Zhang X, Liu Y, Chen J, Hu B, Song J, Zhang Y (2016). Strategies to Optimize Adult Stem Cell Therapy for Tissue Regeneration. Int J Mol Sci.

[14] Cho KA, Park M, Kim YH, Woo SY, Ryu KH (2017). Conditioned media from human palatine tonsil mesenchymal stem cells regulates the interaction between myotubes and fibroblasts by IL-1Ra activity. J Cell Mol Med.

[15] Park M, Kim YH, Ryu JH, Woo SY, Ryu KH (2015). Immune suppressive effects of tonsil-derived mesenchymal stem cells on mouse bone-marrow-derived dendritic cells. Stem Cells Int.

[16] Park M, Kim YH, Woo SY, Lee HJ, Yu Y, Kim HS, Park YS, Jo I, Park JW, Jung SC, Lee H, Jeong B, Ryu KH (2015). Tonsil-derived mesenchymal stem cells ameliorate CCl4-induced liver fibrosis in mice via autophagy activation. Sci Rep.

[17] Cho KA, Park M, Kim YH, Ryu KH, Woo SY (2017). Poly I: C primes the suppressive function of human palatine tonsil-derived MSCs against Th17 differentiation by increasing PD-L1 expression. Immunobiology.

[18] Ritchlin CT, Haas-Smith SA, Li P, Hicks DG, Schwarz EM (2003). Mechanisms of TNF-alpha- and RANKL-mediated osteoclastogenesis and bone resorption in psoriatic arthritis. J Clin Invest.

[19] Pollinger B, Junt T, Metzler B, Walker UA, Tyndall A, Allard C, Bay S, Keller R, Raulf F, Di Padova F, O’Reilly T, Horwood NJ, Patel DD (2011). Th17 cells, not IL-17+ gammadelta T cells, drive arthritic bone destruction in mice and humans. J Immunol.

[20] Chabaud M, Lubberts E, Joosten L, van Den Berg W, Miossec P (2001). IL-17 derived from juxta-articular bone and synovium contributes to joint degradation in rheumatoid arthritis. Arthritis Res.

[21] De Jesus MM, Santiago JS, Trinidad CV, See ME, Semon KR, Fernandez MO, Chung FS (2016). Autologous adipose-derived mesenchymal stromal cells for the treatment of psoriasis vulgaris and psoriatic arthritis: A case report. Cell Transplant.

[22] Zhao K, Lou R, Huang F, Peng Y, Jiang Z, Huang K, Wu X, Zhang Y, Fan Z, Zhou H, Liu C, Xiao Y, Sun J (2015). Immunomodulation effects of mesenchymal stromal cells on acute graft-versus-host disease after hematopoietic stem cell transplantation. Biol Blood Marrow Transplant.

[23] Ryu JH, Park M, Kim BK, Ryu KH, Woo SY (2015). Tonsil-derived mesenchymal stromal cells produce CXCR2-binding chemokines and acquire follicular dendritic cell-like phenotypes under TLR3 stimulation. Cytokine.

[24] Martin TJ, Sims NA (2015). Critical role in bone physiology. Rev Endocr Metab Disord.

[25] Brandtzaeg P (2011). Potential of nasopharynx-associated lymphoid tissue for vaccine responses in the airways. Am J Respir Crit Care Med.

[26] Pabst R (2007). Plasticity and heterogeneity of lymphoid organs. What are the criteria to call a lymphoid organ primary, secondary or tertiary?. Immunol Lett.

[27] Ryu KH, Cho KA, Park HS, Kim JY, Woo SY, Jo I, Choi YH, Park YM, Jung SC, Chung SM, Choi BO, Kim HS (2012). Tonsil-derived mesenchymal stromal cells: evaluation of biologic, immunologic and genetic factors for successful banking. Cytotherapy.

